# Brief Video-Delivered Intervention to Reduce Anxiety and Improve Functioning in Older Veterans: Pilot Randomized Controlled Trial

**DOI:** 10.2196/56959

**Published:** 2024-12-09

**Authors:** Christine E Gould, Chalise Carlson, Julie L Wetherell, Mary K Goldstein, Lauren Anker, Sherry A Beaudreau

**Affiliations:** 1 Geriatric Research, Education and Clinical Center VA Palo Alto Health Care System Palo Alto, CA United States; 2 Department of Psychiatry & Behavioral Sciences Stanford University School of Medicine Stanford, CA United States; 3 Mental Health Impact Unit 3 VA San Diego Healthcare System San Diego, CA United States; 4 Department of Psychiatry University of California, San Diego San Diego, CA United States; 5 Department of Health Policy Stanford University School of Medicine Stanford, CA United States; 6 Sierra Pacific Mental Illness Research, Education, and Clinical Center VA Palo Alto Health Care System Palo Alto, CA United States

**Keywords:** guided self-management, internet, progressive relaxation, technology, anxiety, telehealth, veterans, older adults

## Abstract

**Background:**

Older veterans with anxiety disorders encounter multiple barriers to receiving mental health services, including transportation difficulties, physical limitations, and limited access to providers trained to work with older persons. To address both accessibility and the shortage of available providers, evidence-based treatments that can be delivered via guided self-management modalities are a potential solution.

**Objective:**

This study aims to determine the feasibility and acceptability of a randomized controlled trial of 2 guided self-management interventions. This study compared the treatment effects of these 2 interventions (relaxation and health psychoeducation) on anxiety symptom severity and functioning in older veterans with anxiety disorders. Our exploratory aims examined factors related to home practices and treatment engagement and perceptions of the practices.

**Methods:**

Participants were randomized to one of two video-delivered interventions: (1) Breathing, Relaxation, and Education for Anxiety Treatment in the Home Environment (BREATHE)—breathing and progressive relaxation or (2) Healthy Living for Reduced Anxiety—psychoeducation about lifestyle changes. Telephone coaching calls were conducted weekly. Measures of anxiety, depression, and functioning were obtained at baseline, week 4 (end of treatment), week 8, and week 12. Participants completed a semistructured interview at week 12. Analyses included descriptive statistics to summarize measures of intervention engagement; mixed-effects models to characterize symptom change, and qualitative analyses.

**Results:**

Overall, 56 participants (n=48, 86% men; n=23, 41% from ethnic or racial minority groups; mean age 71.36, SD 6.19 y) were randomized. No difference in retention between study arms was found. The Healthy Living group (29/56, 52%) completed significantly more lessons (mean 3.68, SD 0.86) than the BREATHE group (27/56, 48%; mean 2.85, SD 1.43; *t*_53_=2.60; *P*=.01) but did not differ in completion of coaching calls. In the BREATHE group, greater baseline anxiety scores (*r*=–0.41; *P*=.03) and greater severity of medical comorbidity (*r*=–0.50; *P*=.009) were associated with fewer completed practices. There was no effect of intervention on change in total anxiety scores or functioning. For specific anxiety subtypes, Healthy Living produced a greater decline in somatic anxiety compared with BREATHE. Qualitative analyses found barriers to practicing, including difficulty setting time aside to practice, forgetting, or having other activities that interfered with BREATHE practices. Some participants described adapting their practice routine to fit their daily lives; some also used relaxation skills in everyday situations.

**Conclusions:**

These findings suggest that a larger randomized controlled trial of guided self-management approaches to treating late-life anxiety is feasible; however, BREATHE was not effective in reducing anxiety compared with Healthy Living. Possible contributing factors may have been the reliance on a single technique. Progressive relaxation was reported to be enjoyable for most participants, but maintaining home practices was challenging. Those with milder anxiety severity and fewer health problems were better able to adhere to practices.

**Trial Registration:**

ClinicalTrials.gov NCT02400723; https://clinicaltrials.gov/study/NCT02400723

## Introduction

### Background

Anxiety disorders are pervasive among older adults and especially common in older military veterans. These disorders include generalized anxiety disorder (GAD), social anxiety disorder, panic disorder, agoraphobia, and unspecified anxiety disorders [1]. A meta-analytic review estimated that nearly 1 in 10 (9.1%) older military veterans met the criteria for one of these anxiety disorders [2]. Not only do late-life anxiety symptoms and disorders contribute to detrimental outcomes such as functional and cognitive decline [3-5], but the presence of an anxiety disorder (ie, GAD) is a risk factor for suicide ideation among older non–combat-exposed veterans [6,7]. Thus, access to mental health services for anxiety in older veterans is critical.

Numerous barriers impede older veterans with anxiety from receiving mental health services. These barriers include mental health stigma and beliefs, lack of knowledge about available mental health services, mobility limitations, transportation challenges, and residing in rural areas [8]. Another barrier is the impact of anxiety diagnoses on referral for treatment. Older veterans with anxiety are more likely to receive nonspecific anxiety disorder diagnoses and less likely to receive mental health services compared with younger veterans who receive specific anxiety diagnoses and more subsequent mental health services [9]. Thus, accessible and brief nonpharmacological interventions for late-life anxiety are needed to address the barriers to accessing mental health care faced by older veterans with anxiety disorders. Furthermore, conclusions from a recent systematic review and meta-analysis suggest that these brief interventions may show promise for reducing anxiety symptoms in older adults [10]. One such brief behavioral intervention, progressive relaxation [11], also known as progressive muscle relaxation (PMR), has been shown to be an efficacious behavioral intervention for the treatment of late-life anxiety [12]. PMR is often included as a component within cognitive behavioral therapy (CBT) for anxiety treatments, as well as being a component of digital mental health interventions such as self-management mobile apps. We focused on this skill and developed a 4-week video-delivered intervention that teaches PMR and diaphragmatic breathing and encourages the application of these skills to help patients engage in activities in which anxiety or other types of stress may arise. Activity engagement was selected as a treatment target as discomfort and distress from anxiety symptoms prompt individuals to use coping strategies such as escape from and avoidance of anxiety-evoking situations in the short term [13]. This process (ie, negative reinforcement) makes it more likely for avoidance to be relied upon in the long term and is particularly detrimental to older patients because reduced engagement in activities often leads to isolation and functional decline [7].

This intervention—Breathing, Relaxation, and Education for Anxiety Treatment in the Home Environment (BREATHE)—is organized with weekly video lessons, daily practice videos, and telephone coaching to encourage adherence to the practices. It was initially tested in a proof-of-concept study comparing the 4-week BREATHE intervention to a waitlist control in older adults with anxiety disorders [14]. The BREATHE intervention was found to be superior to the waitlist control in reducing anxiety, depressive, and somatic symptoms; however, the attrition in BREATHE (35%) warranted further investigation. To address these attrition issues, we sought feedback on BREATHE from older veterans and made iterative revisions to the intervention in a second study [15]. The revised BREATHE intervention was then tested in a small feasibility study with 10 older veterans with anxiety disorders [16] in which BREATHE was found to be feasible and acceptable in that 90% (n=9) completed the intervention and 100% felt that BREATHE somewhat or completely met their expectations. While BREATHE is a guided self-management approach to anxiety, it differs from other similar approaches that either rely on manuals and bibliotherapy or are entirely internet delivered (see the study by Cremers et al [17] for a review). BREATHE falls between these 2 approaches, uses familiar technology (web-based videos or DVDs), and focuses on a single skill (PMR) rather than multiple skills (eg, CBT).

### Objectives

In this study, we conducted a pilot randomized controlled trial (RCT) comparing 2 guided self-management interventions in older veterans with anxiety disorders. This study was conducted in part to determine the feasibility of conducting an RCT by documenting willingness to be randomized, engagement with the interventions assigned, and dropout. We also examined the preliminary efficacy of BREATHE compared with a psychoeducation intervention (Healthy Living for Reduced Anxiety) on anxiety symptoms and functioning in older veterans. We hypothesized that (1) BREATHE would result in greater reduction in anxiety symptoms compared with psychoeducation at 12 weeks and (2) BREATHE would result in significantly greater improvements in functioning compared with psychoeducation at 12 weeks. Our exploratory aims were to examine whether home practices and treatment engagement were related to patient characteristics or to intervention outcomes and examine participants’ perceptions of the home practices via qualitative interviews.

## Methods

A 2-group pilot RCT (ClinicalTrials.gov NCT02400723) was conducted over the course of 12 weeks. The interventions lasted 4 weeks, with data collected at baseline, week 4 (end of treatment), week 8 (4 weeks after treatment), and 12 weeks (8 weeks after treatment).

### Ethical Considerations

This study was reviewed and approved by the Stanford University Institutional Review Board (IRB-32454), the institutional review board of record for the US Department of Veterans Affairs (VA) Palo Alto Health Care System. Informed consent was obtained at the baseline visit. Participants were paid US $60 for the initial assessment and the 12 week assessment. Participants were paid US $10 for completing the telephone assessments at weeks 4 and 8. Data were deidentified prior to data entry and analysis.

### Recruitment

Participants were recruited from a large VA health care system via posted flyers and brochures. Informational letters were also sent to patients who had at least one encounter during the past year that was related to a diagnosis of anxiety. Recruitment took place from February 2019 to March 2020 (before the COVID-19 pandemic) and from July 2020 to November 2020 (after the COVID-19 pandemic). Participants were eligible if they were aged ≥60 years and proficient in English and exhibited a diagnosis of an anxiety disorder (ie, GAD, panic disorder, agoraphobia, social anxiety disorder, or unspecified anxiety disorder). Participants were excluded if they were currently enrolled in other intervention research studies or were currently involved in individual therapy or group therapy more frequently than once per month. Those with possible cognitive impairment per a brief cognitive assessment or those who reported a diagnosis of bipolar disorder, psychotic disorder, schizophrenia, or other serious psychiatric disorders were excluded during the telephone screening process. If participants were taking psychotropic medications, they needed to be on a stable dose for 1 month before enrollment.

### Measures

The following sections describe measures for inclusion and exclusion criteria assessment, outcome assessment, and measurement of covariates.

#### Assessment of Inclusion and Exclusion Criteria

The Short Blessed Test (SBT), derived from the Blessed Orientation-Memory-Concentration Test, was included as a brief cognitive assessment to ascertain possible cognitive impairment [18]. Participants obtaining scores of ≥6 on the SBT were excluded from the study [18,19]. The Structured Clinical Interview for the DSM-V (SCID-5 [20]), was administered to assess mental health diagnoses and exclude participants with serious psychiatric disorders, as mentioned previously. Those participants exhibiting an anxiety disorder were included if they met all other inclusion criteria.

#### Demographic Questionnaire

A questionnaire was administered at baseline to assess basic demographic, employment, and health information. Additional questions inquired about participants’ previous experience with relaxation, breathing training, meditation, tai chi, and any other similar techniques.

#### Anxiety Measures

The Geriatric Anxiety Scale (GAS [21]) is a 30-item measure of anxiety that served as a primary outcome measure. The first 25 items measure the frequency of somatic, cognitive, and affective anxiety symptoms and are summed to obtain a total score. The last 5 items assess specific anxiety and fear content and are not included in the total score. Items are scored on a scale from 0 (*not at all*) to 3 (*all of the time*), with total scores ranging from 0 to 75; higher scores indicate greater anxiety. The GAS and its somatic, cognitive, and affective symptom subscales have good internal consistency and convergent validity compared with other anxiety measures [22]. The GAS was administered at baseline, week 4, week 8, and week 12.

The Hamilton Anxiety Rating Scale (HAM-A [23]) assesses the severity of anxiety using clinician ratings of 14 items on a 5-point scale and was included as a secondary outcome measure of anxiety. It has adequate internal consistency, high interrater reliability, and good to adequate concurrent validity [24,25]. The HAM-A was administered at baseline and week 12.

The Patient-Reported Outcomes Measurement Information System 7-item anxiety scale assesses the frequency of experiencing anxiety symptoms within the previous week [26]. Psychometric support for this measure has been found in adult populations [27,28] and in our previous work [16]. The Patient-Reported Outcomes Measurement Information System anxiety scale was administered at baseline and week 12.

The Anxiety Control Questionnaire (ACQ [29]) is a 30-item self-report measure assessing one’s perceived ability to control anxiety-evoking situations and emotional reactions to these situations. Gerolimatos et al [30] found good internal consistency in older adults. The ACQ was administered at baseline and week 12.

#### Functioning

The Activity Card Sort (ACS) [31] was selected as a measure of activity engagement as it assesses the presence and the loss of activities using 80 photographs that depict instrumental, leisure, and social activities. We used an interactive sorting task to calculate lifestyle-adjusted function, which excludes activities that people have never performed in their lifetime [32]. The lifestyle-adjusted function is the number of easy activities divided by the sum of easy activities, hard activities, and no-longer-performed activities. Thus, the lifestyle-adjusted function score accurately reflects loss, gain, or changes in activity participation. This measure was obtained at baseline and week 12. Due to this being an in-person task, the lifestyle-adjusted function scores were only obtained from participants who completed the study before the COVID-19 pandemic.

We also used an individualized scoring of the ACS [33]. In this approach, we asked participants to select 5 activities that they would like to do more frequently if not experiencing anxiety. We ascertained participants’ goals for the number of times they would like to do the activity, and we obtained the frequency of performing the activity at baseline, week 4, week 8, and week 12.

The Veterans RAND 12-item Health Survey (VR-12) [34] examines health-related quality of life and generates 2 scores: a physical component summary and a mental component summary (MCS). The MCS was used as a functioning outcome measure. The VR-12 was administered at baseline, week 4, week 8, and week 12.

#### Additional Measures

The 9-item Patient Health Questionnaire (PHQ-9 [35]) was used to assess participants’ depression symptoms. The questionnaire asks about symptoms during the previous 2 weeks, and each item is scored from 0 (*not at all*) to 3 (*nearly every day*), with total scores ranging from 0 to 27. This measure was administered at baseline and week 12.

The Cumulative Illness Rating Scale–Geriatric [36] measures medical illness burden. Retrospective chart review was used to obtain the ratings and drew on recorded history, physical examination, and laboratory tests, consistent with previous work [37,38].

A semistructured interview and brief survey about the BREATHE or Healthy Living intervention was administered to participants at week 12. The survey included a question in which participants ranked the intervention components (video lessons, practices, and coaching calls) from most helpful to least helpful. The semistructured interview encompassed 7 questions about different domains, including changes made in one’s life as a result of the intervention, effects on one’s well-being, changes in activities and function, when improvement was first noticed, sustainability of the practices, recommended changes to the intervention, and whether the intervention would be recommended to other patients. Herein, we focus on questions related to the home practices in the BREATHE participants.

### Procedures

#### Telephone Screening and Baseline Assessment

Participants completed a brief telephone screen to assess for potential cognitive impairment using the SBT, concurrent psychotherapy, presence of serious mental illness, or recent changes to psychotropic medications (if taking any). Eligible participants based on the telephone screen were invited to a baseline assessment that included obtaining informed consent. Baseline assessments were conducted in person up to February 2020 and via telephone after the onset of the COVID-19 pandemic. During the baseline assessment, a structured psychiatric interview was conducted to ascertain the presence of a current anxiety disorder (ie, GAD, panic disorder, agoraphobia, social anxiety disorder, or unspecified anxiety disorder). Participants who did not meet the criteria for an anxiety disorder or individuals who had a potential psychotic disorder or bipolar disorder were excluded at baseline. The remainder of the assessment consisted of completing the clinical interviews (SCID-5 and HAM-A), ACS, and questionnaires (demographics and health questionnaire, GAS, PHQ-9, ACQ, and VR-12).

#### Randomization

Eligible participants were randomized to BREATHE or psychoeducation in a blocked randomization scheme with blocks of varying sizes (2 to 8), as recommended by the statistician who created the randomization scheme. Each assignment was concealed in an envelope that a research team member opened in sequential order at the time of randomization.

#### Interventions

##### BREATHE Intervention

In the BREATHE intervention, participants watched 1 lesson video each week and then were instructed to practice relaxation 1 to 2 times a day. Participants were able to select whether they viewed the videos from a DVD or from a website. Participants also received weekly calls from their BREATHE coaches during which adherence was ascertained (ie, the lesson video viewed and practices completed). Anxiety ratings for each practice were reviewed, questions were addressed, and encouragement for practice adherence was provided. During weeks 2 to 4, patients were instructed to apply the skills during everyday life. In total, 2 team members (CG and LA) with a master’s to PhD level of training in psychology served as coaches for both the BREATHE and Healthy Living interventions. Coaches used a coaching manual (available upon request) for each intervention and questions tailored to each week’s content to guide the coaching calls. Both BREATHE and Healthy Living coaches addressed any participant challenges with using the DVD or website. Table 1 provides an overview of the intervention components, including the mean duration of the completed coaching calls.

**Table 1:** Summary of intervention components.

Week	BREATHE^a^ components	Healthy Living components
1	Video lesson: “What is anxiety? Diaphragmatic breathing & progressive relaxation” (37 min 35 s) Video-guided daily practice: 16-muscle group relaxation (20 min 50 s) Coaching call: mean 10 min, SD 4 min 42 s	Video lesson: “What is anxiety?” (18 min 45 s) Readings: “Coping with Stress and Aging” and “Stress: Causes and Effects” Coaching call: mean 7 min, SD 3 min 29 s
2	Video lesson: review 16-muscle group relaxation Video-guided daily practice: 16-muscle group relaxation (20 min 50 s) Skill application: use breathing in daily life Coaching call: mean 11 min, SD 4 min 50 s	Video lesson: “Coping with Stress and Aging” (19 min 27 s) Readings: “Sleep Tips” and “Age Page: A Good Night’s Sleep” Coaching call: mean 10 min, SD 5 min 24 s
3	Video lesson: brief relaxation (7-muscle group; 19 min 31 s) Video-guided daily practice: 7-muscle group relaxation (11 min 32 s) Skill application: use progressive relaxation in daily life Coaching call: mean 12 min, SD 4 min 42 s	Video lesson: “Taking care of your body through physical activity” (31 min 20 s) Readings: “Age Page: Exercise and Physical Activity” and “Getting Fit for Life” Coaching call: mean 7 min, SD 4 min 49 s
4	Video lesson: review brief relaxation Video-guided daily practice: 7-muscle group relaxation (11 min 32 s) Skill application: use progressive relaxation in daily life Coaching call: mean 14 min, 7 min 42 sec	Video lesson: “Taking Care of Your Body Through Healthy Eating” (19 min 30 s) Readings: “Healthy Eating & Physical Activity Across Your Lifespan” and “What’s on your plate?” (optional) Coaching call: mean 8 min, SD 3 min, 46 s

^a^BREATHE: Breathing, Relaxation, and Education for Anxiety Treatment in the Home Environment.

##### Psychoeducation (Healthy Living for Reduced Anxiety)

In the psychoeducation intervention (ie, active control), participants viewed 30-minute video lessons once a week for 4 weeks. The videos provided information about what anxiety is, coping with anxiety and sleep tips, benefits of exercise and a gentle stretching routine, and healthy eating. A Healthy Living coach (see the aforementioned description) called participants each week to ascertain adherence (ie, the lesson video viewed) and answer specific questions about the materials. Practice assignments consisted only of brief supplemental readings (Table 1).

#### Assessments at Weeks 4, 8, and 12

At weeks 4 and 8, a total of 4 questionnaires were completed with participants via phone: the GAS, PHQ-9, VR-12, and activity goal frequency (ACS). At week 12 (8 weeks after completion of the 4-week intervention), participants returned for a posttreatment assessment. The posttreatment assessment included the GAS, PHQ-9, HAM-A, ACS, VR-12, and ACQ. A feedback survey for the BREATHE and Healthy Living conditions was completed followed by a semistructured interview. The survey asked participants to rank the helpfulness of each part of the program: video lessons, practices for BREATHE or readings for Healthy Living, and coaching calls. Then, the survey had 5 statements with which participants rated their agreement on a Likert-type scale ranging from *strongly disagree* (1) to *strongly agree* (5). The items asked about the usability of the DVDs, website log-in, and watching of web-based videos and about the frequency of coaching calls and duration of the program. The semistructured interview was recorded with participants’ permission, and the recordings or notes (if not recorded) were then transcribed. During the COVID-19 pandemic, the posttreatment assessment was completed by telephone. Assessors were not blinded to participant condition.

### Power

An a priori sample power analysis was calculated with an α of .05 and power of 0.80. On the basis of previous research, the controlled effect size (Hedges *g*) for relaxation therapy’s effect on anxiety was 0.90 (95% CI 0.44-1.44) [12]. The total estimated sample size was 30 (15 per group; Cohen *f=*0.41). Because of expected attrition and use of self-directed treatment, we estimated a smaller effect of the primary measure of anxiety and, thus, aimed for a sample of at least 26 participants per group (*f=*0.35).

### Statistical Analysis

Descriptive statistics were used to help characterize the sample; *t* tests (2-tailed) and chi-square analyses were used to test whether the 2 groups differed on any characteristics. Correlation analyses were conducted to examine whether baseline anxiety or medical comorbidity were associated with homework completion. Analyses were conducted using SPSS Statistics (version 29.0; IBM Corp) [39].

To examine the hypotheses regarding the primary outcomes of anxiety (GAS) and functioning (VR-12 MCS), mixed-effects models, also known as linear growth models or multilevel models [40], were used. Missing data points due to participant dropout were handled assuming that the data were missing at random and conditional on observed information. Mixed models were used to examine the change in outcomes across 4 time points (baseline [T1], 4 weeks and end of treatment [T2], 8 weeks [T3], and 12 weeks [T4]). Growth models with just time were estimated first followed by the fully specified models that included a between factor of treatment group (BREATHE vs psychoeducation); a within factor of time; and an interaction of treatment by time, which was the estimate of the treatment effect over the course of the study.

A total of 3 mixed-effects models with 2 time points (baseline [T1] and 12 weeks [T4]) were conducted on (1) anxiety as measured using the HAM-A, (2) perceived anxiety control as measured using the ACQ, and (3) lifestyle-adjusted functioning using the ACS. Sensitivity analyses were conducted to evaluate the change in anxiety symptoms (GAS) from baseline to 4 weeks.

Rapid qualitative analysis [41,42] was used to investigate perceptions of the home practices among the participants assigned to the BREATHE intervention. Interview transcripts were summarized using templates, and then domain summaries were created by 2 authors trained in qualitative techniques (CG and CC). The summaries were reviewed for themes.

## Results

### Overview

**Figure 1:**
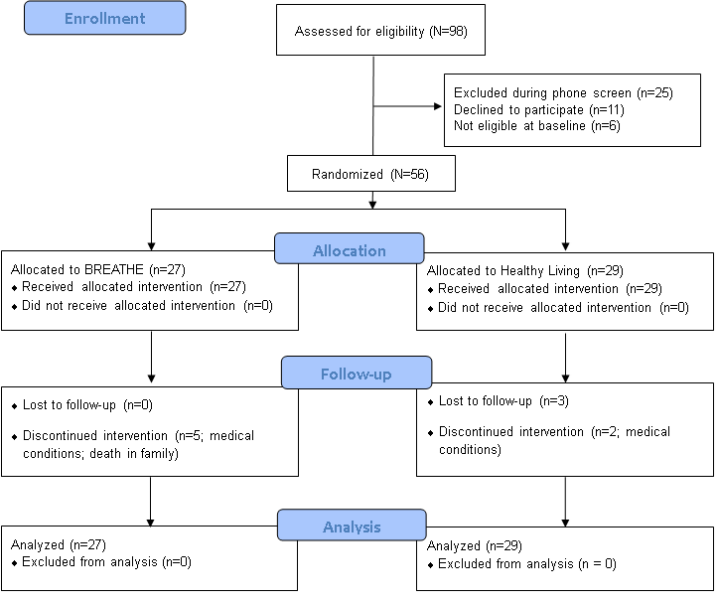
CONSORT (Consolidated Standards of Reporting Trials) flow diagram. BREATHE: Breathing, Relaxation, and Education for Anxiety Treatment in the Home Environment.

Of the 98 participants assessed for eligibility, 56 (57%) were eligible and were all subsequently randomized. Figure 1 shows the participant flow throughout the study.

Randomized participants had a mean age of 71.36 (SD 6.19) years and ranged in age from 60 to 88 years. Table 2 shows the characteristics of participants at baseline. Participants were diagnosed with a current anxiety disorder at baseline using the SCID-5. A total of 16% (9/56) of the participants had more than one concurrent anxiety disorder, 29% (16/56) had co-occurring depression, and 14% (8/56) had subthreshold posttraumatic stress disorder (PTSD; other specified trauma disorder). The most frequently occurring disorders were other specified anxiety disorder (30/56, 54%), GAD (18/56, 32%), social anxiety disorder (11/56, 20%), agoraphobia (2/56, 4%), and panic disorder (2/56, 4%).

**Table 2:** Baseline characteristics of randomized participants (N=56)^a^.

Characteristic			Total		BREATHE^b^ (n=27)		Healthy Living (n=29)		Test of significance							
									*t* test (*df*)			Chi-square (*df*)			*P* value	
Age (years), mean (SD)			71.4 (6.2)		72.8 (6.2)		70.0 (6.0)		–1.73 (54)			—^c^			.09	
Education years, mean (SD)			15.6 (3.0)		15.8 (3.2)		15.5 (2.7)		–0.48 (53)			—			.63	
CIRS-G^d^ medical comorbidity, mean (SD)			13.2 (4.6)		13.8 (5.4)		12.6 (3.8)		–1.01 (53)			—			.32	
**Gender, n (%)**										—			1.2 (2)			.56
	Men	48 (86)		23 (85)		25 (86)										
	Women	7 (12)		3 (11)		4 (14)										
	Nonbinary	1 (2)		1 (4)		0 (0)										
**Race and ethnicity, n (%)**										—			4.1 (4)			.40
	Asian	1 (2)		0 (0)		1 (3)										
	Black	7 (12)		5 (19)		2 (7)										
	Hispanic, any race	10 (18)		5 (19)		5 (17)										
	White	33 (59)		16 (59)		17 (59)										
	Other or multiracial	5 (9)		1 (4)		4 (14)										
**Marital status, n (%)**										—			8.0 (4)			.09
	Single	7 (12)		6 (22)		1 (3)										
	Married	24 (43)		10 (37)		14 (48)										
	Separated or divorced	21 (38)		8 (30)		13 (45)										
	Widowed	4 (7)		3 (11)		1 (3)										
**Employment status, n (%)**										—			1.8 (3)			.61
	Working full time	6 (11)		2 (7)		4 (14)										
	Working part time	5 (9)		2 (7)		3 (10)										
	Unemployed	4 (7)		3 (11)		1 (3)										
	Retired	41 (73)		20 (74)		21 (72)										
**Health status, n (%)**										—			2.7 (4)			.61
	Excellent	5 (9)		3 (11)		2 (7)										
	Very good	8 (14)		3 (11)		5 (17)										
	Good	23 (41)		12 (44)		11 (38)										
	Fair	18 (32)		9 (33)		9 (31)										
	Poor	2 (4)		0 (0)		2 (7)										
**Psychotropic medication, n (%)**										—			0.0 (1)			.85
	Yes	31 (55)		15 (56)		16 (55)										
	No	24 (43)		11 (41)		13 (45)										
	Missing	1 (2)		1 (4)		0 (0)										
**Previous psychological counseling, n (%)**										—			0.0 (1)			.83
	Yes	43 (77)		20 (74)		23 (79)										
	No	12 (21)		6 (22)		6 (21)										
	Missing	1 (2)		1 (4)		0 (0)										
**Previous complementary health experience, n (%)**																
	Meditation	10 (18)		7 (26)		3 (10)		—			2.3 (1)			.13		
	Yoga	5 (9)		2 (7)		3 (10)		—			0.2 (1)			.70		
	Tai chi	4 (7)		1 (4)		3 (10)		—			0.9 (1)			.34		
	Regular exercise	17 (30)		6 (22)		11 (38)		—			1.9 (1)			.17		

^a^No significant differences were found for the Breathing, Relaxation, and Education for Anxiety Treatment in the Home Environment and Healthy Living participants. Fisher exact tests confirmed chi-square results in variables with <5 per cell.

^b^BREATHE: Breathing, Relaxation, and Education for Anxiety Treatment in the Home Environment.

^c^Not applicable.

^d^CIRS-G: Cumulative Illness Rating Scale–Geriatric.

### Retention and Engagement

Completion of the assessment at 4 weeks and after treatment was 81% (22/27) for BREATHE and 93% (27/29) for Healthy Living (χ^2^_2_=1.7; *P=*.19). The retention at week 12 was similar across groups, with 81.5% (22/27) of BREATHE participants and 82.8% (24/29) of Healthy Living participants completing the assessment at 12 weeks (T4). The groups did not differ with regard to which modality they used for the delivery of the videos (χ^2^=2.4; *P*=.30). Across both groups, 66% (37/56) used DVD delivery, 21% (12/56) used web delivery, 7% (4/56) used both, and 5% (3/56) had missing data on modality due to dropout from the study.

Most of the weekly coaching calls were completed for both interventions, with no differences found in the number of weekly calls completed for each intervention (t_54_=0.92; *P*=.36). On average, BREATHE participants completed 3.48 (SD 1.05) of 4 (87%) weekly coaching calls. Healthy Living participants completed 3.72 (SD 0.92) of 4 (93%) weekly coaching calls.

With regard to engagement with the intervention as self-reported to coaches, the Healthy Living group completed significantly more lessons (mean 3.55, SD 1.09) than the BREATHE group (mean 2.85, SD 1.43; t_54_=2.07; *P*=.04). In addition to the weekly lessons, the BREATHE group had daily home practices and 3 assignments to apply the skills in real life. Pearson correlation analyses found that greater baseline anxiety scores (GAS) were associated with fewer completed practices in the BREATHE group (*r*=–0.41; *P*=.03). In addition, greater severity of medical comorbidity (Cumulative Illness Rating Scale–Geriatric) was associated with fewer completed practices in BREATHE (*r*=–0.50; *P*=.009). On average, BREATHE participants completed 23.07 (SD 17.38) practices during the 4-week intervention. A total of 22% (6/27) of the participants completed 1 application of the skills, 19% (5/27) completed 2 applications of the skills, and 30% (8/27) completed ≥3 applications.

Participants shared feedback about the interventions through a postsurvey (Table 3). Participants generally agreed to strongly agreed regarding the ease of use and the usability of the DVD and website to access the videos. Most participants agreed to strongly agreed that the coaching calls were frequent enough and the program length was sufficient.

**Table 3:** Intervention feedback survey^a^.

Survey item	BREATHE^b^, mean (SD)	Healthy Living, mean (SD)	Test of significance	
			*t* test (*df*)	*P* value
The DVDs were easy to use.^c^	4.67 (0.69)	4.35 (0.93)	1.18 (36)	.25
The log-in for the website was easy.^d^	4.17 (0.72)	4.79 (0.43)	–2.72 (24)	.01
It was easy to watch the videos on the website.^e^	4.33 (0.71)	4.69 (0.63)	–0.13 (20)	.23
The calls from my coach were frequent enough.^f^	4.30 (0.66)	4.21 (0.83)	0.40 (42)	.69
The program was the right length of time.^f^	4.25 (0.79)	4.29 (0.75)	–0.18 (42)	.86

^a^Responses ranged from *strongly disagree* (1) to *strongly agree* (5). Due to some participants choosing to use DVDs and others using web delivery, the number of responses to the postsurvey question varied.

^b^BREATHE: Breathing, Relaxation, and Education for Anxiety Treatment in the Home Environment.

^c^BREATHE: n=18; Healthy Living: n=20.

^d^BREATHE: n=12; Healthy Living: n=14.

^e^BREATHE: n=9; Healthy Living: n=13.

^f^BREATHE: n=20; Healthy Living: n=24.

### Primary and Secondary Analyses

Treatment effects were examined using mixed-effects models conducted on total anxiety scores as measured using the GAS. The models were best fitted with a fixed effect of time and group and random intercept. Table 4 shows the means for the BREATHE and Healthy Living groups across 4 time points (baseline [T1], 4 weeks and end of treatment [T2], 8 weeks [T3], and 12 weeks [T4]). Notably, there was substantial variability for baseline GAS scores, with some participants reporting low symptoms in the previous week despite meeting the criteria for an anxiety disorder. There were no differences in the rates of decline in anxiety between the groups. That is, the effects of time (*P*=.07), group (*P*=.64), and interaction of treatment by group (*P*=.07) were not significant. An inspection of the subscales revealed that there was a significant time by group interaction for somatic anxiety symptoms (*F*_3, 145.6_=2.81; *P*=.04) but not for affective or cognitive anxiety symptoms. This significant interaction indicated that the Healthy Living group experienced a significant decline in somatic anxiety over time compared with the BREATHE group.

**Table 4:** Means and SDs for outcome measures at 4-week intervals.

			BREATHE^a^, mean (SD)		Healthy Living, mean (SD)		Test of significance		
							*t* test (*df*)		*P* value
**Baseline^b^**									
	GAS^c^	17.07 (10.35)		18.79 (10.50)		0.62 (54)		.54	
	GAS somatic	7.37 (4.33)		7.31 (4.75)		–0.05 (54)		.96	
	GAS cognitive	4.56 (3.67)		5.41 (3.31)		0.92 (54)		.36	
	GAS affective	5.15 (4.29)		6.07 (3.87)		0.84 (54)		.40	
	PHQ-9^d^	7.37 (6.07)		6.69 (4.64)		–0.47 (54)		.64	
	VR-12^e^ MCS^f^	16.07 (3.10)		14.93 (3.83)		–1.22 (54)		.23	
**End of treatment (week 4)^g^**									
	GAS	17.86 (10.04)		14.11 (9.51)		–1.35 (48)		.18	
	GAS somatic	8.22 (4.06)		5.61 (4.11)		–2.08 (48)		.04	
	GAS cognitive	4.73 (3.49)		3.67 (2.93)		–1.15 (48)		.25	
	GAS affective	5.00 (4.19)		4.54 (3.50)		–0.26 (48)		.80	
	PHQ-9	7.64 (5.80)		5.14 (4.27)		–1.75 (48)		.09	
	VR-12 MCS	16.00 (3.55)		14.04 (3.31)		–2.02 (48)		.05	
**Week 8^h^**									
	GAS	14.19 (6.46)		14.73 (11.35)		0.19 (45)		.85	
	GAS somatic	7.10 (3.73)		6.15 (5.03)		–0.71 (45)		.48	
	GAS cognitive	3.43 (2.79)		4.04 (4.05)		0.59 (45)		.56	
	GAS affective	3.67 (2.33)		4.54 (3.50)		0.98 (45)		.33	
	PHQ-9	6.86 (5.35)		6.27 (6.53)		–0.33 (45)		.74	
	VR-12 MCS	15.10 (3.13)		13.65 (3.24)		–1.54 (45)		.13	
**Week 12^i^**									
	GAS	18.41 (10.96)		15.67 (9.19)		–0.92 (44)		.36	
	GAS somatic	8.64 (4.74)		6.58 (4.03)		–1.59 (44)		.12	
	GAS cognitive	4.64 (3.16)		4.04 (3.63)		–0.59 (44)		.56	
	GAS affective	5.14 (4.44)		5.04 (2.79)		–0.09 (44)		.93	
	PHQ-9	6.92 (5.66)		5.96 (4.62)		–0.63 (44)		.53	
	VR-12 MCS	15.82 (3.42)		13.42 (3.82)		–2.24 (44)		.03	

^a^BREATHE: Breathing, Relaxation, and Education for Anxiety Treatment in the Home Environment.

^b^BREATHE: n=27; Healthy Living: n=29.

^c^GAS: Geriatric Anxiety Scale.

^d^PHQ-9: 9-item Patient Health Questionnaire.

^e^VR-12: Veterans RAND 12-item Health Survey.

^f^MCS: mental component summary.

^g^BREATHE: n=22; Healthy Living: n=28.

^h^BREATHE: n=21; Healthy Living: n=26.

^i^BREATHE: n=22; Healthy Living: n=24.

No difference was found with regard to a difference in BREATHE or Healthy Living effect on mental functioning over time as measured using the VR-12. In addition, no difference was found between treatment groups with regard to depressive symptoms as measured using the PHQ-9 (Table 4), anxiety symptoms as measured using the HAM-A, lifestyle-adjusted functioning as measured using the ACS, or anxiety control as measured using the ACQ (Table 5).

**Table 5:** Means and SDs for outcome measures at baseline and week 12^a^.

			BREATHE^b^, mean (SD)		Healthy Living, mean (SD)		Test of significance		
							*t* test (*df*)		*P* value
**Baseline^c^**									
	ACS^d^ lifestyle-adjusted function^e^	42.66 (14.05)		45.65 (13.81)		0.74 (45)		.47	
	PROMIS^f^ anxiety	17.74 (6.91)		18.97 (6.40)		0.69 (54)		.49	
	ACQ^g^	91.48 (23.87)		84.24 (21.00)		–1.21 (54)		.22	
	HAM-A^h^	18.04 (7.57)		16.59 (7.10)		–0.74 (54)		.46	
**Week 12^i^**									
	ACS lifestyle-adjusted function^j^	46.30 (13.02)		49.77 (15.51)		0.69 (31)		.50	
	PROMIS anxiety^k^	17.64 (6.74)		16.25 (6.47)		–0.71 (44)		.48	
	ACQ	97.96 (30.06)		91.08 (21.97)		–0.91 (46)		.37	
	HAM-A^k^	15.55 (7.30)		15.54 (7.17)		–0.002 (44)		.99	

^a^With the exception of the Activity Card Sort and Anxiety Control Questionnaire, higher scores indicate worse symptom severity. On the Activity Card Sort, higher scores indicate a greater percentage of activities that are easy to do. On the Anxiety Control Questionnaire, higher scores indicate greater perceived anxiety control. Due to missing data, the number of participants at baseline varied.

^b^BREATHE: Breathing, Relaxation, and Education for Anxiety Treatment in the Home Environment.

^c^BREATHE: n=27; Healthy Living: n=29.

^d^ACS: Activity Card Sort.

^e^BREATHE: n=23; Healthy Living: n=24.

^f^PROMIS: Patient-Reported Outcomes Measurement Information System.

^g^ACQ: Anxiety Control Questionnaire.

^h^HAM-A: Hamilton Anxiety Rating Scale.

^i^BREATHE: n=23; Healthy Living: n=25.

^j^BREATHE: n=22; Healthy Living: n=23.

^k^BREATHE: n=15; Healthy Living: n=18.

### Effects at Week 4 and Association With Engagement Measures

As shown in Table 4, the BREATHE and Healthy Living groups did not differ in effects at week 4 and end of treatment with the exception of GAS somatic scale scores, which were lower for Healthy Living compared with BREATHE at this time point. We also investigated whether changes in scores from baseline to week 4 were associated with home practice completion. Using Pearson correlation analyses, we found the association with practice completion to be nonsignificant (*r*=0.33; *P=*.13). Reduction in GAS scores from baseline to week 4 (end of treatment) was not associated with completion of lessons (*r_s_*=–0.09; *P*=.53) or coaching calls (*r_s_*=–0.009; *P*=.95) across the BREATHE and Healthy Living combined samples.

### Individual Activity Goals

Individual activity goals set by participants were examined with regard to the number of goals identified, types of goals, and change in individualized goal frequencies from baseline to 4 weeks. On average, participants selected at least 4 activities to focus on (mean 4.7, SD 1.2; range 2-7). Of 263 total activities identified, the 3 most frequently selected activity categories were social activities (72/263, 27.4%); high-demand leisure activities (70/263, 26.6%) such as hiking or swimming; and low-demand leisure activities (63/263, 24%) such as photography, reading, or playing a musical instrument. The remaining activities were instrumental activities (35/263, 13.3%) or other activities identified by participants (23/263, 8.7%).

Change scores for each activity goal were calculated from 0 to 4 weeks. Scores were then collapsed into 2 categories: increase in activity frequency (change score of >0) or no change or decrease in activity frequency (change score of ≤0). The BREATHE and Healthy Living groups did not differ in their distribution of increased activities at 4 weeks (χ^2^=0.2; *P*=.64). A total of 79% (23/29) of Healthy Living participants and 74% (20/27) of BREATHE participants reported an increase in frequency of one or more goals at 4 weeks. On average, participants in both groups attained ≥1 goal at 4 weeks (BREATHE: mean 1.6, SD 1.4; Healthy Living: mean 1.6, SD 1.3; consistent with an intention-to-treat approach, all participants were retained in these analyses. Those individuals who were lost to follow-up were coded as not attaining their goal).

### Qualitative Feedback Regarding Practice Routine and Challenges

Feedback regarding the experience of the practices in the BREATHE group, including the overall experience of the practices, mechanics and frequency of practices, challenges encountered, and application of skills to real-world situations, was analyzed to better understand participants’ experience with the BREATHE intervention (22/27, 81% completed the interviews). While Healthy Living participants relayed the same type of information, in this section, we focus on the BREATHE intervention as the condition of interest.

#### Experience With Practices

Participants expressed varied experiences with the practice components of the BREATHE intervention, that is, the diaphragmatic breathing and the PMR exercises. Overall, most participants ranked the practices as the most (9/21, 43%) or second (9/21, 43%) most helpful component of the intervention compared with the weekly coaching calls and video lessons. Some described the diaphragmatic breathing exercises as the most effective part of the intervention and the easiest skill to use outside of home. One participant (BR1) noted the following:

The breathing definitely was important. That’s probably a big part of it. The breathing practices, because I didn’t normally do this before, but by doing breathing exercises, it definitely helped me.

Participants described adjustments made to PMR exercises. These included tensing for less time (BR12); adjusting for physical issues (BR18), which could include imagined tensing as specified in the videos; and grouping some muscles together (eg, lower extremities and facial muscles; BR18). Another participant liked tensing from the waist up (ie, face and upper torso) “because I get a lot of tension there” (BR4). A smaller number of participants felt that the practices and skills were not helpful in that breathing would not solve the problems they were facing. One individual explained the following:

I don’t think learning how to breathe a certain way is gonna make anything go away that I’m dealing with.

BR5

Another participant noted that tensing their muscles was not helpful “because I’m tense already” (BR3).

#### Mechanics and Frequency of Practices

The mechanics of how and when people practiced varied. Creating a practice habit through timing, frequency (eg, once or twice a day), or location seemed to help with accomplishing the practices without stress or feeling pressured. One participant described the following:

...that the only time that I could do this was lying down—was in bed, lying down. And I thought—Oh, gosh, it’s not going to be as effective. But after life quieted down somewhat, I was able to attain my goal and start to do it twice a day. Attempt to do it twice a day. But it was still lying down, and I loved it.

BR8

For some, setting a schedule to practice was stressful and, instead, practicing during a block of time (eg, mornings and evenings) helped:

It’s like I like to do stretching exercises every day and I would kind of keep myself physically fit and this was more kind of a mental exercise of imposing discipline on myself to be able to do this twice a day.

BR6

With regard to the frequency of practice, participants described practicing once or twice a day for the first month or 2; however, many participants described that their practice frequency decreased after the first 4 weeks, which coincided with the weekly check-in calls ending after the first 4 weeks. For some participants, the relaxation procedures may have been too static (ie, the same video used to guide practice), and after several weeks, this contributed to diminished engagement. In contrast, some described returning to the practices to help with anxiety. One participant explained the following:

I didn’t do it [progressive relaxation] as much for a while. And then out of necessity I said, “This is what I need to do,” and I have done it more in the past months.

BR16

Another individual expressed an intention to continue practicing:

I will keep doing this ’cause I enjoy setting aside a time for a disciplined approach to trying to relax.

BR6

Personalization of the practices seemed to facilitate continued use of the techniques. One participant described adjusting their practice over time:

I changed it from watching the videos to just actually sitting, closing my eyes, and going through the process. I felt most comfortable doing it that way.

BR1

The guidance helped one participant “keep my mind on track” (BR8).

#### Challenges

Participants identified several challenges with completing the practices. Setting time aside to practice daily was a challenge due to timing, forgetting (BR2 and BR15), traveling (BR7), having visitors (BR6), or other activities. One participant described the following:

So, carving out the time is a little bit of a challenge. It’s not overwhelming, because the program is really not that demanding, really, when you think about the time. But you just get busy.

BR2

Health and other life challenges deprioritized the study for some participants (BR5). Technology difficulties were noted by 9% (2/22) of the participants, and 5% (1/22) encountered difficulties, but described learning the technology. Documenting anxiety scores was identified as tedious by another individual.

#### Application of Skills in the Real World

Participants described multiple applications of the relaxation and breathing practice in the real world. The most common applications were using the skills when experiencing anxiety, tension, or stress or whenever a situation dictated it. Some described noticing tense or anxious feelings and then using the practices to alleviate the symptoms:

...when I began to feel periods of anxiety, I would either go to immediately and practice, or I’d set time aside [to practice].

BR10

Others described specific situations in which they applied the skills, including dentist appointments (BR4), at the hospital (BR9), or when having an operation (BR11). Some used the breathing or tensing and releasing tools while driving and in traffic or while waiting for VA appointments (eg, BR8 and BR13). Another veteran described deep breathing when a task became more complicated or frustrating:

What helps me is that if I’m sitting there making breakfast and I see that it’s getting complicated, something is frustrating me that maybe I dropped something that immediately I will just close my eyes and breathe slow. And sometimes I can only breathe three and then, if I feel I need it, I just go into doing more. And it’s not a big conversation, I just do it.

BR16

## Discussion

### Principal Findings

The findings of this pilot RCT of BREATHE compared with Healthy Living demonstrate the feasibility of conducting an RCT in that all participants were willing to be randomized and retention was similar between study arms at 12 weeks. Some variation in engagement with and acceptability of the interventions emerged, as discussed in the following sections, but does not decrease the feasibility of conducting an RCT. The hypotheses were not supported with regard to the greater reduction in total anxiety symptoms or improvement in functioning (as measured using the VR-12 MCS score) in BREATHE compared with the psychoeducation control of Healthy Living. On the anxiety symptom subscales, participants in the Healthy Living condition experienced a greater reduction in somatic anxiety symptoms, but no other differences were found for the other 2 subscales of cognitive and affective anxiety symptoms. There are several reasons for these findings. First, as observed in most previous late-life intervention studies [43], the inclusion of an active control may have diminished the potential effect of relaxation. Furthermore, a growing body of research has focused on the importance of well-being–focused interventions, including yoga [44], nutrition [45], and other alternative interventions [46], and the presence of some of these factors may have contributed to the benefits that participants achieved in the Healthy Living intervention. This intervention did include gentle stretching and some basic sleep and coping tips in addition to nutritional information, which all may relate to physical and mental health while aging. It is possible that a single-component intervention was not effective for engaging participants across a 4-week period. Recent studies have demonstrated that CBT for depression and anxiety is effective at not only reducing anxiety but also preventing relapse over a 10-year period [47]. In addition, the heterogeneity of anxiety disorders, as detected using structured interviews, and the range in anxiety severity based on baseline GAS scores may lead to attenuated findings with a single-component intervention rather than a multicomponent intervention to address varied facets of anxiety (ie, cognitive, somatic, and affective symptoms).

Our exploratory aim examined factors related to home practices in the BREATHE condition or related to intervention outcomes which revealed that greater self-reported anxiety symptom severity and greater medical comorbidity at baseline were associated with fewer progressive relaxation practices completed during the 4-week intervention. The qualitative findings helped us further probe the potential effect of practices. Qualitative findings suggested that, for some, practicing breathing, relaxation, or both breathing and relaxation was not viewed as enough to manage their anxiety and stress, thus dovetailing with the quantitative finding of those participants with greater anxiety practicing less. Accordingly, BREATHE or Healthy Living may be better suited for individuals with mild to moderate anxiety, which should be tested directly in a future study. It is possible that older veterans with greater distress would have preferred having access to group or individual psychotherapy and may have had previous exposure to these modalities as a function of the integration of mental health into the Veterans Health Administration [48].

Other challenges to practicing were recounted by participants, including difficulty finding the time to practice, having other things to do (eg, visitors and other activities), or worsening of health problems and experiencing acute health changes. Some participants reported a decline in practices after the coaching calls ended at the 4-week mark. Thus, the challenges with practices were 2-fold and included both adhering to the practices and maintaining a practice routine over time, which parallels the challenges of having sustained engagement with digital mental health interventions [49] and with any behavioral intervention requiring practice. Perhaps improvements and variations in the types of relaxation are needed to maintain interest in practices over time.

These findings led to the question of whether home practice is the issue itself. Integrating an ongoing relaxation practice into daily life while facing multiple medical conditions may have required extra effort and motivation from participants. The qualitative interview findings revealed that participants were able to personalize their practices based on their own needs, tailor their routine, and implement the relaxation procedures in their daily life when they needed it. Another possible future direction would be to provide multiple skills in a modular approach consistent with digital mental health interventions [50] or consider adding a session focused on motivational interviewing to the intervention.

Participants described using the skills in a myriad of settings, including when facing worries or anxiety, when in a stressful situation, or when simply waiting for an appointment, and they achieved one or more goals on average. Future studies should clarify the dose of PMR needed and whether preferences for psychotherapy compared with technology [51] play a role in practice completion and overall adherence. Alternatively, while these interventions were designed to be widely accessible and scalable, it is possible that a more mechanistic consideration of breathing approaches for specific individuals may be needed (ie, precision medicine approaches [52]). In addition, more engaging and adaptive technology–based interventions could be of benefit for late-life anxiety and lead to more sustained engagement with practices. Further refinement of goal setting to promote intervention engagement alongside skills to cope with anxiety is needed. On average, BREATHE and Healthy Living participants attained at least one goal at the end of treatment, but this could be strengthened using more directed, individualized outcome measures for the goals (eg, goal attainment scaling approaches [53]).

### Limitations

This study is not without limitations. One of the key limitations may be the heterogeneity of anxiety disorders and symptom presentation in a relatively small pilot study. Rather than including multiple types of anxiety disorders, focusing on specific symptoms (eg, worry that exceeds a particular threshold on a questionnaire) may be a better approach, as used with other studies [44,54]. As this sample included veterans, we likely encountered higher rates of co-occurring PTSD compared with other nonveteran samples. This particular comorbidity of PTSD may have made the anxiety presentation more complex and difficult to treat using guided self-management interventions. Additional limitations include the small sample size as the study was designed to be a pilot, the use of nonblinded assessors, and the absence of a validated measure of acceptability. Another limitation to our design was that only a portion of the outcome measures were assessed at the end of treatment (T2). This study took place earlier in the COVID-19 pandemic, which led to some modifications in data collection methods and limited our ability to collect complete data, in particular from the lifestyle-adjusted ACS.

### Comparison With Prior Work

These findings differ from those of earlier studies on progressive relaxation for late-life anxiety in that the effects of progressive relaxation were not as robust as those found in earlier studies [55]. The use of a primarily male, ethnically and racially diverse military veteran sample may have contributed to some differences compared with earlier studies, in which older nonveteran White women made up much of the samples. The benefits derived from the Healthy Living intervention fit with the evidence for complementary and integrated health approaches for late-life anxiety [44] and the Whole Health program in the Veterans Health Administration [56].

### Conclusions

These findings suggest that guided self-management approaches to treating late-life anxiety in older veterans are feasible but that further refinement and study are needed to identify what works best for whom using a video-delivered format with remote coaching. Our findings suggest that a psychoeducation-based approach may help older adults with somatic anxiety symptoms. While progressive relaxation was deemed to be feasible and enjoyable to most participants, those with mild to moderate anxiety symptom severity and fewer health problems were more likely to adhere to the recommended home practice. Thus, based on the qualitative feedback, the BREATHE intervention in particular might not be a good match for all older adults with anxiety disorders due to some participants needing a higher-intensity treatment and others experiencing negative reactions to progressive relaxation, primarily “relaxation-induced anxiety” [57]. Further work is needed to delineate the role of intervention design factors and individual participant baseline characteristics on the effect of guided self-management approaches on late-life anxiety.

## Appendix

Multimedia Appendix 1 CONSORT-eHEALTH checklist (V 1.6.1).

## Abbreviations

ACQ: Anxiety Control Questionnaire
ACS: Activity Card Sort
BREATHE: Breathing, Relaxation, and Education for Anxiety Treatment in the Home Environment
CBT: cognitive behavioral therapy
GAD: generalized anxiety disorder
GAS: Geriatric Anxiety Scale
HAM-A: Hamilton Anxiety Rating Scale
MCS: mental component summary
PHQ-9: 9-item Patient Health Questionnaire
PMR: progressive muscle relaxation
PTSD: posttraumatic stress disorder
RCT: randomized controlled trial
SBT: Short Blessed Test
SCID-5: Structured Clinical Interview for the DSM-V
VA: US Department of Veterans Affairs
VR-12: Veterans RAND 12-item Health Survey
